# Longitudinal ambulatory measurements of gait abnormality in dystrophin-deficient dogs

**DOI:** 10.1186/1471-2474-12-75

**Published:** 2011-04-13

**Authors:** Inès Barthélémy, Eric Barrey, Pablo Aguilar, Ane Uriarte, Matthias Le Chevoir, Jean-Laurent Thibaud, Thomas Voit, Stéphane Blot, Jean-Yves Hogrel

**Affiliations:** 1 UPR de Neurobiologie, Ecole Nationale Vétérinaire d'Alfort, 94704 Maisons-Alfort, France; 2 INRA, Génétique animale et biologie intégrative, 78352 Jouy-en-Josas, France; 3 Unité de biologie intégrative des adaptations à l'effort, INSERM 902, 91000 Evry, France; 4 Institut de Myologie, Université Paris 6 UMR S974, INSERM U974, CNRS UMR 7215, GH Pitié-Salpêtrière, 75651 Paris Cedex 13, France

## Abstract

**Background:**

This study aimed to measure the gait abnormalities in GRMD (Golden retriever muscular dystrophy) dogs during growth and disease progression using an ambulatory gait analyzer (3D-accelerometers) as a possible tool to assess the effects of a therapeutic intervention.

**Methods:**

Six healthy and twelve GRMD dogs were evaluated twice monthly, from the age of two to nine months. The evolution of each gait variable previously shown to be modified in control and dystrophin-deficient adults was assessed using two-ways variance analysis (age, clinical status) with repeated measurements. A principal component analysis (PCA) was applied to perfect multivariate data interpretation.

**Results:**

Speed, stride length, total power and force significantly already decreased (p < 0.01) at the age of 2 months. The other gait variables (stride frequency, relative power distributions along the three axes) became modified at later stages. Using the PCA analysis, a global gait index taking into account the main gait variables was calculated, and was also consistent to detect the early changes in the GRMD gait patterns, as well as the progressive degradation of gait quality.

**Conclusion:**

The gait variables measured by the accelerometers were sensitive to early detect and follow the gait disorders and mirrored the heterogeneity of clinical presentations, giving sense to monitor gait in GRMD dogs during progression of the disease and pre-clinical therapeutic trials.

## Background

Duchenne muscular dystrophy (DMD), a still incurable severe and generalized muscle disease in man, represents a therapeutic challenge because of the numerous obstacles it presents for systemic gene, cell or pharmacological therapies. Several of these issues, such as efficacy of studied drugs, or locoregional or systemic medication pathways, can be assessed in the dystrophin-deficient dog, a large animal model which mimics the human disease in many points, better than other animal models [1].

During pre-clinical trials, to assess a functional effect, these canine patients must undergo a clinical follow-up encompassing a qualitative and quantitative evaluation of their impaired functions, particularly the gait. Recently, two studies including ours have focused on gait analysis in adult dogs suffering from Golden retriever muscular dystrophy (GRMD), using two different tools [2,3]. Both studies have demonstrated that GRMD dogs walk slower than healthy controls, and that several specific gait parameters were altered. The kinematic analysis of the hind limb has shown an increase of the stifle joint extension, and a decrease of the hock joint flexion, this last joint being moreover less mobile than in control dogs [2]. The accelerometry study we have conducted has shown less regular and powerful accelerations, a decrease of the stride length and frequency, and a redistribution of the power from the cranio-caudal to the medio-lateral axis [3].

These studies have demonstrated that gait analysis is feasible and repeatable in adult GRMD dogs and that these animals exhibit quantifiable gait impairments. However, the majority of the treatments that are tested in GRMD dogs are administered during the first months of life, at a period when the morphological changes due to growth as well as the clinical evolution rate are maximal [4-6]. Therefore, we sought to longitudinally characterize the dynamic evolution of the gait parameters tested by an ambulatory gait analysis system based on 3D-accelerometers, in order to delineate the disease progression in the GRMD model over time. We aimed to address the question if the gait variables are able to reflect disease evolution according to the previous results obtained in control and dystrophin-deficient adults [3]. Another question was to determine if the gait analysis was able to quantitatively describe the well-known heterogeneity of the model [1,7]. These points represent pre-requisites for the use of this method in pre-clinical trials. This is the first longitudinal study on gait analysis in growing GRMD dogs, and the first study using an ambulatory gait analysis system to monitor the canine gait evolution, during growth and disease progression.

## Methods

### Subjects

All procedures were carried out in accordance with the *Guide of the Care and the Use of Laboratory Animals*, and approved by the Ethical Committee of the National Veterinary School of Alfort.

Six healthy golden retriever and twelve GRMD male dogs were included in the study. All the dogs were housed in the same facilities. The healthy dogs were littermates of some GRMD dogs of the study. Two of them (Ckan and Cbof) have taken part in the first study at an adult age [3]. The dogs were genotyped as previously described [8], before the age of 2 months, and GRMD dogs were randomly selected among the newborns of the French GRMD colony. Each GRMD dog underwent the clinical score evaluation monthly during the whole study period, using our previously described grid [9]. In our grading system, the more the dog is affected, the higher the score is. No GRMD dog had taken part in the previous study on adult dogs [3].

### Materials

The 3-dimensional accelerometer recorder used in this study was a Locometrix^® ^gait analysis system, composed of 3 orthogonally-positioned accelerometers, to allow the recording of the accelerations along the dorso-ventral, cranio-caudal and medio-lateral axes of the dogs, as previously described [3]. The measurement range of the accelerometer was ± 2 g, with a resolution of 0.001 g. The signal was recorded at a sampling rate of 100 Hz and anti-aliasing filter was used with a cut off set at 50 Hz.

### Gait testing

As previously described [3], dogs were carried from the kennel to a 45 metre-long corridor located close to the laboratory facilities. The belt in which the accelerometric device was inserted was fastened around the thorax of the dog, so that the device was placed under the sternum, near to the centre of gravity at rest.

The study design encompassed 15 tests per animal, at a frequency of one test session twice monthly, from the age of two months, that can be considered as the onset of motor clinical signs in animals surviving to the neonatal form, to nine months, i.e. during the period of growth and of disease progression [1]. A few minutes before the first test at two months, the young puppies were familiarized to the corridor and to the belt. The height of each dog on the back, immediately caudally to the shoulders, defining the height at withers (HW), was measured at the end of each test. All the tests were performed by the same operator (IB).The test was performed as previously described, encouraging the dog to walk or run at his preferred gait and speed, and timing him over five metres for each used gait [3]. The free ambulation choice has been previously argued in studies on GRMD locomotion [2,3], and will be further discussed.

### Data analysis

The analysis of the acceleration curves was performed using the Equimetrix^® ^software for quadrupedal gait analysis. Every sequence of 10 seconds of steady state locomotion was analysed, whatever the type of gait, which was easily identifiable on the dorso-ventral acceleration curves (Additional file 1). The speed measured during the test for each given type of gait was used for the analysis. The regularity, an index quantifying the similarity of dorso-ventral accelerations over successive strides, was used as an indicator of quality of gait, in order to select the gait the dog would be more comfortable with. Thus, when more than one type of gait (walk, trot or gallop) was analyzed during a test, the type of gait for which the regularity score was the best was selected.

The studied variables were described in details in previous publications [3,10]. Except for the speed, which was measured by the operator during the test, over 5 metres of steady gait, they were all calculated by the adapted software Equimetrix^® ^for quadrupedal locomotion analysis, as follows:

• The stride frequency (/s), calculated as a quarter (walk) or a half (trot, gallop) of the fundamental frequency, using Fast Fourier Transform (FFT).

• The stride length (m), calculated by dividing the speed by the stride frequency.

• The stride regularity (dimensionless), calculated by adding two coefficients of correlation (correlation of acceleration within a stride, and between strides), obtained by calculating the autocorrelation function on the dorso-ventral acceleration signal. The sum of these two coefficients was multiplied by 100 and normalized by a Z transform.

• The total power of accelerations (W/kg), calculated by adding the powers calculated in the three axes. The power in each direction was computed as the integral of the power spectrum obtained by the FFT from the raw acceleration signal.

• The relative components of the total power along the three axes (%), calculated by dividing the cranio-caudal, dorso-ventral or medio-lateral power by the total power.

• The relative force index (N/kg), calculated by dividing the total power by the speed, in order to avoid variations due to different speeds, and/or to different types of gait. As 1 W = 1 Nm.s^-1^, the resulting ratio (W.kg^-1^/m.s^-1^) was expressed in N/kg, and named relative force index.

The speed and the stride length were normalized by the height at withers (HW), in order to avoid the effect of size on these variables.

### Statistical analysis

For each variable, a repeated measures analysis of variance, with age as a within effect factor and group (GRMD vs. Healthy) as a between effect factor was used in order to assess:

• The effect of age on the evolution of gait variables in Healthy dogs

• The effect of age and disease progression on the evolution of gait variables in GRMD dogs

• The impact of the disease on the evolution of gait variables

• The differences between the two groups

A Pearson's correlation coefficient was calculated in order to study the correlations between variables, and mainly between the clinical score and the gait variables in GRMD dogs.

Finally, in order to summarize the results we proposed a graphic representation of the gait evolution of the dogs. The individuals were projected, as supplementary observations, on the reference PCA plane that we previously defined in [3], using eight healthy and 11 GRMD adult dogs, and the seven following variables: stride length/HW, stride frequency, total power, medio-lateral, cranio-caudal, dorso-ventral parts of the total power, and regularity. For each age point, the Euclidean distance in the seven-dimensional space between each GRMD dog and the centre of gravity of the six age-matched healthy controls was calculated in order to assess if this distance could be used as an overall "gait score" variable, as previously described by Schutte et al. [11]. This distance was also submitted to the repeated measures ANOVA, and a Pearson's correlation coefficient to the clinical score was calculated.

The level of statistical significance was set at p = 0.01 for all the tests.

## Results

### Test compliance

All the planned test sessions could be performed in the six healthy dogs (Table 1). However, at the age of 2.5 months, four of them were too unruly and their acceleration curves too unstable to be analyzed. Therefore, no comparison between both groups was possible at this time point. The preferential gait was the trot at each age point, and for all the dogs, except for three of them at 2 months, and for one of them at 3 months, who performed a rotary gallop.

**Table 1:** Animals included in the study.

		Age point (months)														
		
		2	2.5	3	3.5	4	4.5	5	5.5	6	6.5	7	7.5	8	8.5	9
		
*Healthy*																
Ckan		T	T	T	T	T	T	***na***	T	T	T	***na***	T	T	T	T
Cbof		RG	T	T	T	T	T	***na***	T	T	T	***na***	T	T	T	T
Dclick		T	***na***	T	T	T	T	T	T	T	T	T	T	T	T	T
Dclack		RG	***na***	T	T	T	T	T	T	***na***	T	T	T	T	T	T
Dzer		T	***na***	T	T	T	T	T	T	T	T	T	T	T	T	T
Dzastre		RG	***na***	RG	T	T	T	T	T	T	T	T	T	T	T	T

***GRMD***																

Dchou		T	T	W	W	***Loss of locomotion***										
Dmo		BG	BG	BG	BG	***na***	BG	***Death***								
Dfois		T	T	T	T	W	W	W	***Loss of locomotion***							
Dsir		T	***na***	T	T	T	W	W	***Loss of locomotion***							
Dlire		BG	BG	BG	T	T	BG	T	T	T	T	T	T	W	T	***Death***
Dlice		T	BG	BG	BG	BG	***na***	T	T	T	T	T	W	T	T	W
Dluge		T	T	BG	BG	BG	BG	BG	T	BG	BG	W	T	T	T	BG
Dk		BG	T	BG	W	BG	BG	BG	BG	BG	T	BG	W	W	W	W
Dbrouille		T	T	T	T	T	T	T	T	T	T	T	T	T	T	T
Dmon		BG	T	BG	BG	T	T	T	W	T	T	T	T	W	***na***	W
Dalton		BG	***na***	T	T	T	T	T	T	T	T	T	T	T	T	T
Didon		T	BG	BG	BG	BG	BG	BG	BG	BG	BG	BG	W	W	BG	BG

The six healthy and 12 GRMD dogs included in the study are summarized in this table, as well as the significant clinical events, i.e. those which have led to a premature end of the study in some GRMD dogs. The symbol of each GRMD dog, which is used in the figures, is mentioned beside his name. For each age point and each animal, if the test was successful, the most regular gait, which was selected for the analysis, is mentioned using the following letters: W (walk), T (trot), RG (rotary gallop), BG (bound gallop). The mention ***na ***means that the test was not analysable.

Five of the 12 GRMD dogs died before the age of nine months and therefore could not perform the 15 tests (see Table 1). Among these five dogs, three (Dchou, Dfois and Dsir) were euthanized due to a complete loss of locomotion and permanent recumbency, at the age of four (Dchou) and six (Dfois and Dsir) months. Another dog, Dmo, died from digestive complication of the disease at five months. The last dog, Dlire, developed severe aspiration pneumonia at nine months, and could therefore not perform the last test because of pronounced dyspnea; he died the following day.

Among the seven dogs who completed all the tests, six (all except Dbrouille) were successfully treated for at least one episode of aspiration pneumonia, and four (Dk, Didon, Dlice and Dmon) got a gastrostomy tube when they became unable to feed by themselves and needed a fluid correction to maintain a normal hydration state.

The GRMD dogs employed three different types of gait: the so-called "bunny hopping" bound gallop, the trot and the walk. The different types of gait were easily identifiable on the dorso-ventral acceleration curves (Additional file 1).

**Figure 1:**
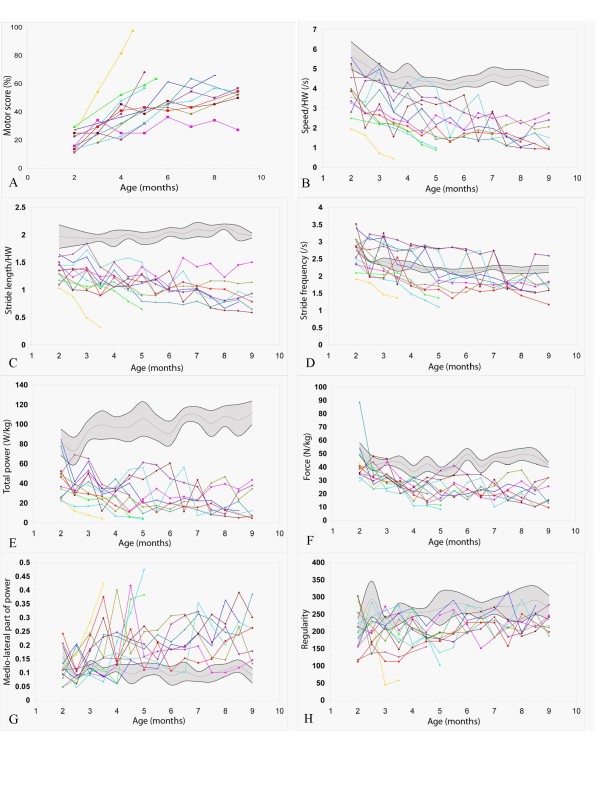
**Evolution of the different gait variables with age **. Each GRMD dog is represented using its own symbol (see table 1). The healthy population is represented by a grey zone, covering the mean (grey line) ± 1 SD (black lines). A: evolution of the motor score in GRMD dogs; B: evolution of the speed normalized by the height at withers (HW); C: evolution of the stride length normalized by the height at withers (HW); D: evolution of the stride frequency; E: evolution of the total power; F: evolution of the force; G: evolution of the medio-lateral part of the power; H: evolution of the regularity.

The main statistical results are presented in Table 2, and details regarding each age point are summarized in Additional file 2. Figure 1 offers a graphic representation of the individual evolutions of GRMD dogs with age, in comparison to the mean ± 1 SD of healthy controls. A ± 1 SD range was chosen because the variables did not always follow a normal distribution, giving poor sense to a ± 2 SD range representation.

**Table 2:** Main results of the statistical analyses.

		Speed (m/s)	Speed/HW (/s)	SL (m)	SL/HW	SF (/s)	TP (W/kg)	Force (N/kg)	CCP/TP (%)	DVP/TP (%)	MLP/TP (%)	Regularity	Distance
Age effect	Healthy	p < 0.0001 (↗)	p = 0.0024 (↘)	p < 0.0001 (↗)	NS	p < 0.0001 (↘)	NS	NS	NS	NS	p = 0.0089 (↘)	p = 0.0145 (↗)	p = 0.0068 (↘)
	GRMD	NS	p = 0.0067 (↘)	NS	NS	p = 0.0350 (↘)	NS	p = 0.0034 (↘)	NS	NS	NS	NS	NS
	Group effect	p < 0.0001	p = 0.0003	p = 0.0002	p = 0.0002	NS	p < 0.0001	p = 0.0006	NS	p = 0.0427	p = 0.0162	p = 0.0090	p < 0.0001

2 months	Healthy	1.77 (0.29)	5.68 (0.90)	0.61 (0.07)	1.97 (0.26)	2.89 (0.20)	81.5 (18.6)	53.2 (6.3)	44.0 (7.7)	40.3 (7.5)	15.8 (6.1)	197 (49)	1.14 (0.60)
	GRMD	1.02 (0.24)	3.72 (1.04)	0.37 (0.09)	1.35 (0.27)	2.70 (0.70)	44.1 (19.2)	43.4 (20.7)	45.7 (9.9)	34.7 (10.7)	11.2 (13.1)	209 (56)	2.61 (0.73)
	p	p = 0.0010	p = 0.0054	p < 0.0001	p = 0.0001	NS	p = 0.0017	NS	NS	NS	NS	NS	p = 0.0031

9 months	Healthy	2.61 (0.18)	4.39 (0.26)	1.18 (0.03)	1.99 (0.06)	2.21 (0.14)	111.4 (16.3)	41.7 (2.6)	47.8 (4.8)	43.3 (3.9)	8.9 (3.5)	269 (44)	0.80 (0.24)
	GRMD	0.88 (0.46)	1.66 (0.75)	0.49 (0.22)	0.92 (0.31)	1.77 (0.43)	20.8 (16.9)	20.4 (9.3)	44.7 (13.5)	29.4 (6.6)	23.8 (9.1)	225 (31)	3.7 (0.93)
	p	p < 0.0001	p < 0.0001	p < 0.0001	p < 0.0001	p = 0.0337	p < 0.0001	p = 0.0002	NS	p = 0.0009	p = 0.0033	NS	p < 0.0001

Correlation with motor score		-0.4353	-0.6480		-0.6834	-0.5082	-0.5640	-0.5368	-0.3492		0.4454		0.6248
	p	p = 0.0002	p < 0.0001	NS	p < 0.0001	p < 0.0001	p < 0.0001	p < 0.0001	p = 0.0030	NS	p = 0.0001	NS	p < 0.0001

For each variable, the age effect for each group is presented in the first row, as well as the group effect. The two following rows show the mean (SD) of healthy dogs/the mean (SD) of GRMD dogs, just above the p value, for the two extreme age points (i.e. 2 and 9 months). The Pearson's coefficient of correlation with the motor score and associated p values are given in the last row. The data at each age point are given in Additional file 2 available online.

Abbreviations: HW: height at withers, SL: stride length, SF: stride frequency, TP: total power, CCP/TP: cranio-caudal part of the total power, DVP/TP; dorso-ventral part of the total power, MLP/TP: medio-lateral part of the total power, Distance (PCA): distance from the centre of gravity of the healthy group on the principal analysis component plane, NS: not significant.

### Evolution of the variables with age in healthy dogs

The six healthy dogs were shown to walk quicker with age (p < 0.0001) and to increase their stride length (p < 0.0001). Speed and stride length were found significantly correlated with height at withers (respective Pearson's coefficients of correlation: 0.78, 0.93; p < 0.0001). When normalized by the height at withers, no age effect on the stride length was identified anymore, whereas the speed was shown to decrease with age (p = 0.0024), probably due to a decrease of the stride frequency (p < 0.0001). The medio-lateral part of the power decreased with age (p < 0.01). No significant age effect was shown for the other variables (i.e. the total power, the cranio-caudal and the dorso-ventral part of the power, the force and the regularity).

### Evolution of the variables with age in GRMD dogs

From the age of 2 months, the gait of GRMD dogs was shown to be significantly (p < 0.001) slower than the gait of healthy dogs, even when the measured speed was normalized by the height at withers (p < 0.01). No age effect on speed could be identified in GRMD dogs, while this variable was found to increase in growing healthy dogs. However, when normalized by the height at withers, the speed significantly decreased with age (p < 0.01) in GRMD dogs.

In the same way, the stride length was already significantly lower in GRMD dogs at the age of 2 months (p < 0.0001), even normalized by the height at withers (p < 0.0001). As for the speed, no age effect on stride length was demonstrated, whereas growing healthy dogs increased their stride length. No age effect was either identified on this variable normalized by the height at withers. However, considering the individual evolutions, it appeared that some dogs tended to decrease their stride length, while others tended to maintain or to increase it.

This inter-individual variability was also striking for the stride frequency parameter. Indeed, some dogs which were able to use a regular typical "bunny hopping" bound gallop gait until a late stage, maintained stride frequency values above those measured in healthy animals, whereas dogs which had switched to trot or walk did less frequent strides. More generally, the lowered stride frequency was found to be a late modification of gait (significant decrease from the age of 7.5 months, p < 0.0001 at this time point). Relative to healthy dogs, GRMD dogs tended to decrease their stride frequency with age, but not significantly (p = 0.017).

Conversely, but similarly to speed and stride length, the total power of the gait was significantly decreased as early as at 2 months of age (p < 0.01). No age effect on the total power evolution in the GRMD population was highlighted, probably due to individual variations in diverging directions.

The relative force index, calculated by dividing the total power by the speed, was shown to be significantly different from the age of 3 months (p < 0.0001), and to decrease with age (p < 0.01). A GRMD dog, Dlice, had a very high force value at 2 months, resulting from one of the best power values of the GRMD group, and from a low speed, this puppy being the smallest of the study at two months.

The relative distribution of the power along the three axes was also shown to be modified in GRMD dogs. At several time points from the age of 3.5 months and until the age of 9 months, a decreased relative dorso-ventral power was shown. The medio-lateral part of the total power was shown to be increased from the age of 5.5 months (p < 0.01). The cranio-caudal part of the power was not modified between 2 and 9 months of age.

The regularity index was significantly decreased only at the 4.5 months time point (p < 0.01). No significant age effect on the evolution of this variable was demonstrated.

Significant correlations with the motor score were found for 8 variables (see Table 2).

**Figure 2:**
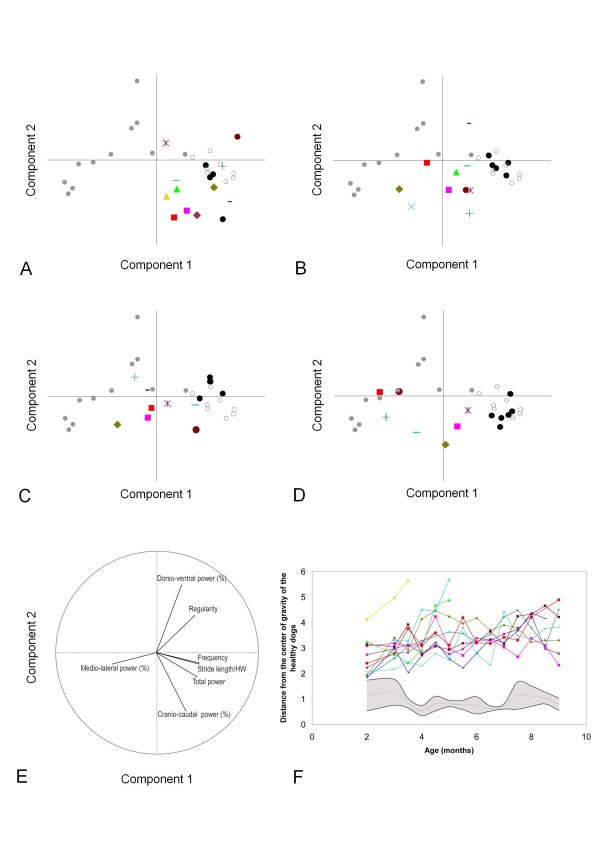
**Using principal component analysis (PCA) for the follow-up of individuals **. The active individuals, used to define the plane, were eight healthy adult dogs represented by empty circles, and eleven GRMD adult dogs represented by grey points. 94.53% of the total variance is explained by the two first components, component 1 along the X axis and component 2 along the Y axis. The projection of the individuals on the PCA plane following these components are represented in panels A to D, and the projection of the variables in panel E. The healthy and GRMD dogs of the present study were used as supplementary individuals. Each GRMD dog is represented using his own symbol (see table 1). The healthy growing dogs are represented by black points. A: projection of the 2 months-old dogs on the PCA plane; B: projection of the 4 months-old dogs on the PCA plane; C: projection of the 6 months-old dogs on the PCA plane; D: projection of the 9 months-old dogs on the PCA plane. E: projection of the seven variables. The first component explains 72.61% of the total variance and is mainly explained by the stride frequency, the stride length, the total power and the medio-lateral part of the power. The second component explains 21.92% of the total variance and is mainly explained by the cranio-caudal and the dorso-ventral part of the power. F Evolution of the distance of each GRMD dog from the centre of gravity of the six age-matched healthy controls. The grey zone represents the mean (grey line) ± 1SD (black lines) of this distance in healthy dogs.

Figure 2 shows the course of healthy and GRMD dogs on the principal component analysis (PCA) plane at 2, 4, 6 and 9 months, and the evolution of the Euclidean distance between the GRMD individuals and the control population with age. The levels of statistical significance of this global gait index are summarized in Table 2. It has to be firstly underlined that the healthy dogs progressively and nicely superimposed as supplementary individuals to the healthy adult population of the reference PCA plane defined in our previous study [3]. If the distance of GRMD dogs from the healthy group is already significantly increased at the age of 2 months, the graphic representation of the growing animals on the PCA plane demonstrated that the GRMD population progressively moves towards the left side, away from the healthy animals, essentially following the component 1 axis, which is mainly explained by the stride length and frequency, the total power and the medio-lateral part of power. At the latest time point, the GRMD dogs were superimposed to the adult GRMD dogs scatter plot, regarding the first component. Their localization along the component 2 axis was however variable, more directed along the cranio-caudal relative power vector than along the dorso-ventral one. The graph showing the evolution of the distance between groups in relation with the age illustrates the progressive separation of both populations, as well as the huge gait quality heterogeneity in the GRMD population, using a quantitative condensed variable calculated from seven accelerometric raw variables. Finally, this distance was found positively correlated to the clinical motor score (R = 0.62, p < 0.0001). However it was obviously able to better distinguish between GRMD dogs: at the age of 9 months for instance, all the GRMD dogs except Dbrouille were found very close to each other using the motor score (Figure 1), whereas a clear separation was identified using the distance from the healthy population on the PCA plane (Figure 2).

## Discussion

This study has demonstrated the ability of 3D-accelerometry to early monitor the gait of GRMD dogs during growth and disease progression. It is the first study quantitatively describing sequential gait impairments in this canine model of DMD.

Early modifications of GRMD dogs' gait have been highlighted, at a stage when the gait could appear normal to a non-experienced observer, attesting to the sensitivity of the accelerometric method. Indeed as soon as at the age of two months, a dramatic lack of power was detected, resulting in a speed decrease, primarily explained by a drop in stride length. The decrease of stride frequency was a later event, secondarily contributing to the reduction of speed. These patterns of evolution are very close to those already described in DMD patients [12,13].

Whatever the age of the dogs, the total power of GRMD dogs was shown to be strongly decreased, but varied with age in its three-axial composition. The decrease in total power was primarily harmonious among the three axes. Then, the relative dorso-ventral power dropped, followed by a later increase of the medio-lateral part of power. This last event has been described in adult GRMD dogs [3], but combined with a decrease of the cranio-caudal part of power, which was not present in growing puppies, even in 9 months-old animals. Some of the growing GRMD dogs were examined at the age of 12 months, and this test showed that within 3 months, the cranio-caudal relative power dropped and the dorso-ventral one normalized (data not shown). Briefly, during the first year of life of GRMD dogs, the gait first becomes less bouncing, maybe as a consequence of the early muscle weakness to support weight, then waddling maybe reflecting contractures and less balance, and finally less propelling and mobile.

One remarkable issue is the wide intra-individual variations of some variables between fortnightly sessions. If the repeatability between sessions has been demonstrated for adult clinically stabilized GRMD dogs [3], the same does obviously not hold true for clinically evolving and growing animals. It may be assumed that these variations are due to the fluctuating day-to-day general shape of the diseased animals during their first months of life. This inter-session variability also underlines the necessity of a careful clinical follow-up of these dogs and of frequent iterative examinations. The simplicity, the lightness and non-invasivity of the accelerometric device allow the repetition of tests over a short period of time.

A methodological issue relies on the fact that the animals self-select their speed of gait. Indeed the measurements give rise to comparisons of variables calculated from acceleration curves recorded over a wide range of speeds and with three types of gait. However, to the experience of teams working on GRMD dogs' locomotion [2,3], this wide range of speed abilities would precisely make a standardisation of speed very difficult, if not impossible. As an example, if a standardized speed had been considered, the lowest speed measured in GRMD adults dogs, i.e. 0.26 m/s [3] should have been chosen, to allow all the ambulant dogs to perform the test. This protocol could not be retained as it is by far artificial and devoid of clinical sense, to force all the dogs to walk at this very low speed. Moreover, the habituation time required by the use of a treadmill on dogs would also probably be significant, and delay the beginning of the follow-up, which should be as early as possible, in a context of therapeutic trials. However, it is undeniable that most of the studied variables are influenced by the speed, and one may put forward that all the modifications observed in GRMD dogs are only due to speed decrease. The decreased relative force index proves that this is not true. This index was created in order to avoid bias due to different speeds on the total power, by dividing it by the speed. It is interesting to note that the evolution of this variable globally follows the evolution of the total power, attesting to both the variations of this variable and the differences with the healthy population are not only due to differences in speed of locomotion. Furthermore, the spontaneous speed of gait is known to be of clinical significance [14,15] and for this reason it represents a relevant measure in GRMD dogs. Finally, one may analyse this issue of speed variations as a limitation, but we assume this can be seen as an advantage as well, because of the clinical pertinence provided by the self-selection of gait and speed. With the same intention to quantitatively assess a situation as spontaneous and physiological as possible, the preferred and more comfortable gait was selected, using the regularity index.

Attesting to the success of this process, the studied variables were, in a vast majority, correlated to the motor score, meaning that the degradation of the gait measured by accelerometry corresponds to the observed clinical evolution. In the same way as the clinical score, the gait variables reveal different evolution types. However, the relative SD observed among the GRMD population for the gait variables are larger than for the motor score (data not shown). This observation supports the argument for a higher sensitivity of the accelerometric measurements variables than the motor score to describe the dogs' locomotion ability. Moreover, if the motor score is a good index to quantify the general shape of the dog, it remains a non-objective evaluation, to use with caution, particularly in non double-blinded studies. However, accelerometry is the first fully objective and quantitative method of gait analysis, successfully assessed in clinically evolving GRMD dogs.

One of the objectives of this study was to assess if a longitudinal follow-up of one particular individual was possible. Examining more closely the results obtained dog per dog, different evolution profiles can be identified, allowing a quantitative description of the well-described heterogeneity of the disease [1,7]. Notably, the three dogs having lost locomotion before the age of six months (Dchou, Dfois and Dsir) share the same characteristics of evolution. Within few weeks, their gait variables reached values comparable to or worse than the values of adult GRMD dogs. Thus, this disease-course bound to result in loss of locomotion, is not only more severe in outcome but also accelerated in its evolution. Moreover, at the first test session at 2 months, the values of stride frequency and of speed measured for these three dogs were among the lowest. This last point suggests that, as early as at the age of 2 months, their gait is already more impaired than the one of dogs who will not lose locomotion. Low stride frequency and speed values at 2 months could be considered as prognostic markers of poor outcome. The predictive value of these biomarkers needs a validation study with more animals. Concerning the 9 other dogs included in the study, different evolution patterns were also identifiable, in function of their capacity to perform a regular and maintained high frequency "bunny hopping" gallop. This "bunny hopping" gallop, also called bound, is used by large size mammals in acceleration and deceleration phases of a classical rotary gallop [16]. In the case of GRMD dogs, the maintenance of bound, a primitive high-frequency gait, could be an adaptive response to muscle weakness, to produce more power using two synchronized limbs, and avoiding a single limb weight support [16]. Two dogs, Dbrouille and Dalton, exhibited surprising evolution patterns. Indeed, after a period of degradation, they tended to improve their gait. This observation emphasizes the need to carefully interpret a slight improvement of gait in an animal participating in a treatment protocol.

In order to simplify the data interpretation, and because of the multitude of variables of interest, we applied the PCA multivariate exploratory method as a tool to position each dog in comparison to the others during time. We have chosen to project the puppies as supplementary observations on the PCA plane defined by the eight healthy and the 11 GRMD adult dogs of our previous study [3]. This has allowed analyzing the chronology of the relative trajectories of healthy and GRMD groups, and locating and following each animal at different ages on a reference PCA plane.

We proposed the use of the Euclidean distance from a control age-matched group as an overall variable to make gait analysis easier regarding data management and interpretation. This distance has already been described and used as a gait analysis index in kinematic studies [11,17]. Our results regarding the evolution of this variable show that it can be used as a global gait index during pre-clinical trials, to improve the readability of the results.

## Conclusion

This longitudinal study has allowed the description of gait pattern evolutions in GRMD dogs at a time when the disease becomes symptomatic, and has identified quantifiable and sequentially impaired gait variables. The ability of accelerometry to monitor gait evolution in dogs during several months, and to quantitatively reflect the heterogeneity of the disease with a good sensitivity has been demonstrated. The PCA analysis was proposed as a final data analysis method, making the interpretation of multivariate data as simple as their measure. Our study showed that a 3D-accelerometric gait analysis device is ready to be used as a simple and reliable gait follow-up tool during pre-clinical therapeutic trials, providing reliable biomarkers quantitatively showing the heterogeneity of the canine GRMD disease, a characteristic to take into account when evaluating this pre-clinical model, and which represents another feature in common with DMD.

## Competing interests

EB was the inventor of the gait analysis method based on accelerometry (Locometrix and Equimetrix). This method was patented by the *Institut National de Recherche Agronomique *(INRA). The method has been validated in human medicine and animal locomotion studies. Other authors have no competing interest.

## Authors' contributions

IB designed and performed the experiments, analyzed the data and drafted the manuscript. EB conceived and designed the experiments, analyzed the data and drafted the manuscript. PA, AU and MLC performed the experiments. JLT performed the experiments and drafted the manuscript. TV drafted the manuscript. SB and JYH designed the experiments, analyzed the data and drafted the manuscript. All authors read and approved the final version of the manuscript.

## Pre-publication history

The pre-publication history for this paper can be accessed here:

http://www.biomedcentral.com/1471-2474/12/75/prepub

## Supplementary Material

Additional file 1 **Aspect of the dorso-ventral acceleration curves of the different types of gait observed in healthy and GRMD dogs**. Samples of 10.24 seconds. A: Types of gait in healthy dogs. B: Types of gait in GRMD dogs. Click here for file

Additional file 2 **Statistical results at the studied age points**. Click here for file
