# Supplementary material for: Longitudinal ambulatory measurements of gait abnormality in dystrophin-deficient dogs
Source: BMC Musculoskelet Disord. 2011 Apr 13;12:75. doi: 10.1186/1471-2474-12-75 (PMC3103492; doi:10.1186/1471-2474-12-75)

A. Healthy dogs

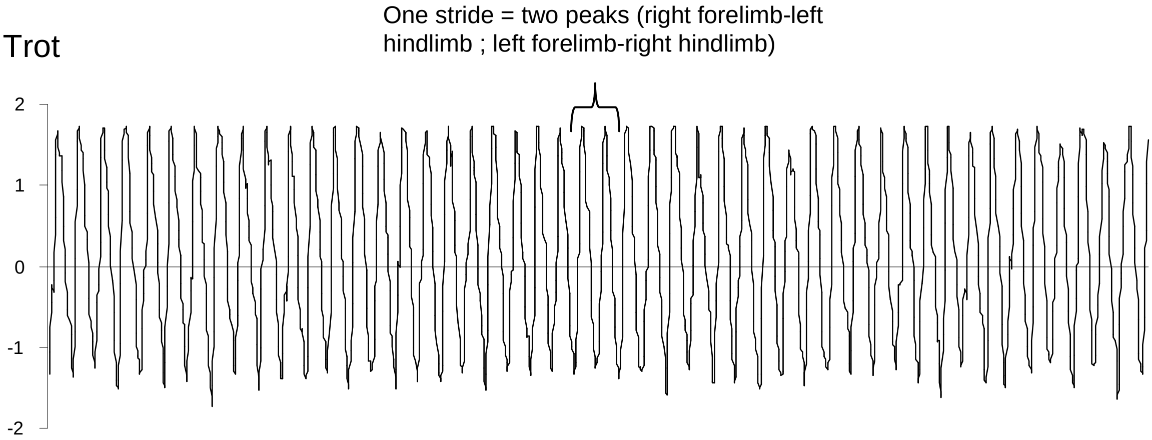

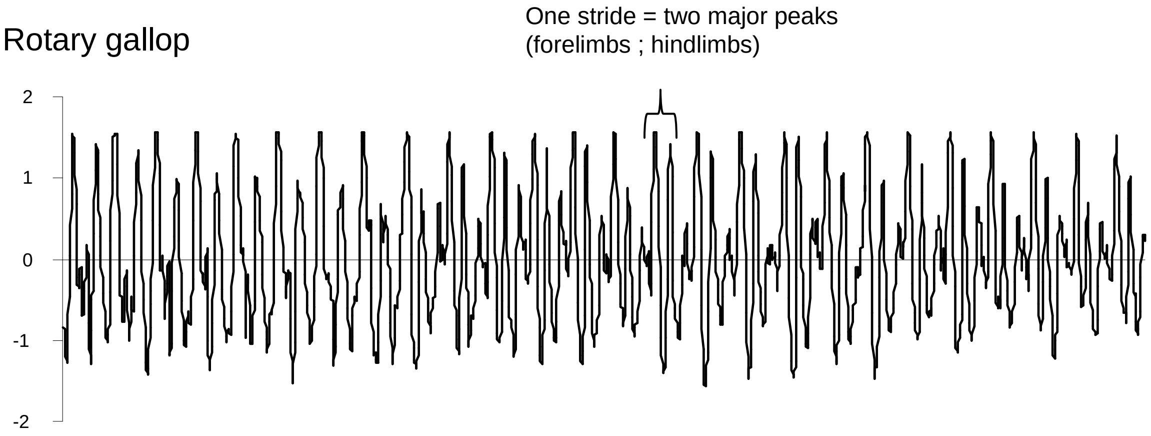

B. GRMD dogs

Bound gallop

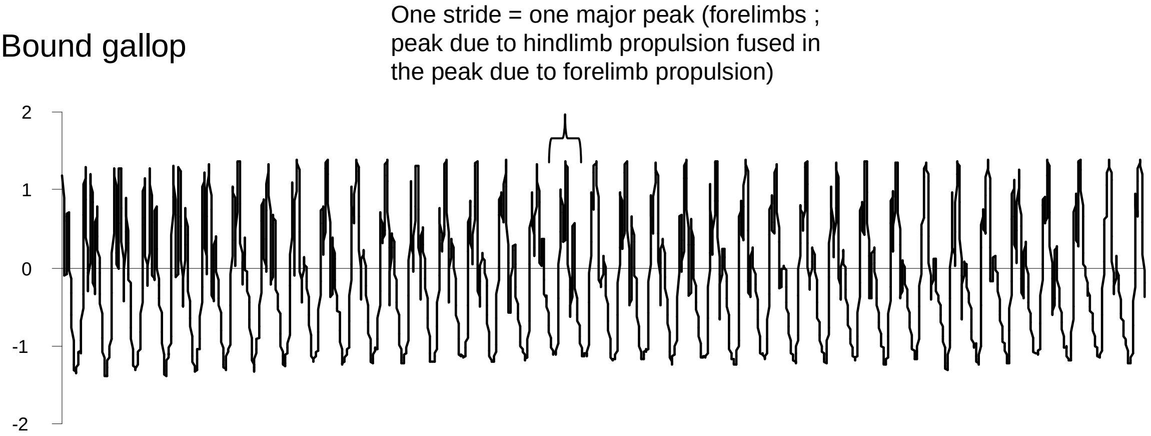

Trot

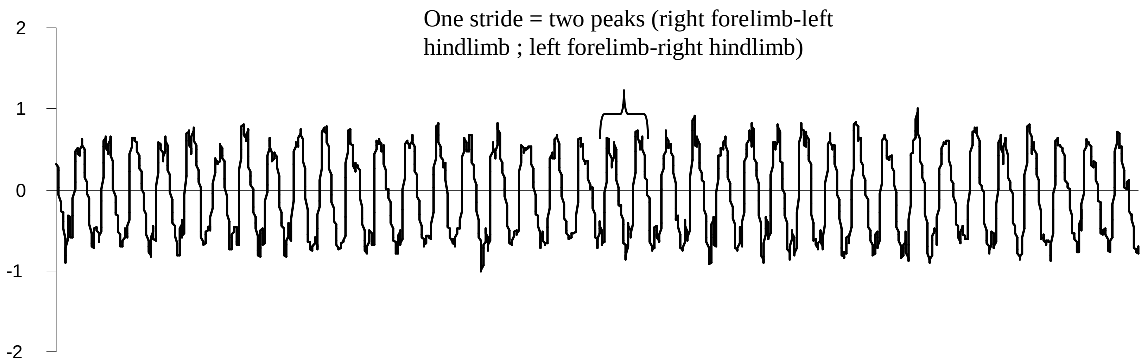

Walk

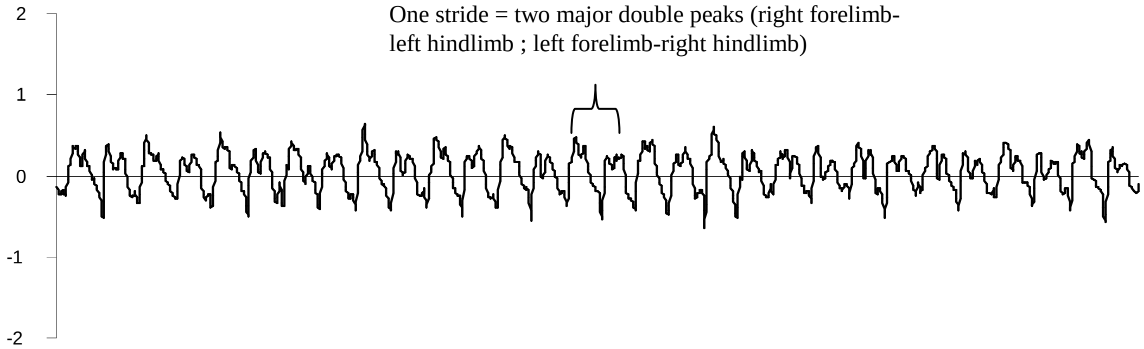

Supplement: Additional file 1 — Aspect of the dorso-ventral acceleration curves of the different types of gait observed in healthy and GRMD dogs. Samples of 10.24 seconds. A: Types of gait in healthy dogs. B: Types of gait in GRMD dogs. [file 1471-2474-12-75-S1.PDF]
