# Supplementary material for: Longitudinal ambulatory measurements of gait abnormality in dystrophin-deficient dogs
Source: BMC Musculoskelet Disord. 2011 Apr 13;12:75. doi: 10.1186/1471-2474-12-75 (PMC3103492; doi:10.1186/1471-2474-12-75)
Supplement: Additional file 2 — Statistical results at the studied age points. [file 1471-2474-12-75-S2.PDF]

Age (months)		Speed (m/s)	Speed/HW (/s)	SL (m)	SL/HW	SF (/s)	TP (W/kg)	Force (N/kg)	CCP/TP (%)	DVP/TP (%)	MLP/TP (%)	Regularity	Distance
2	Healthy	1.77 (0.29)	5.68 (0.90)	0.61 (0.07)	1.97 (0.26)	2.89 (0.20)	81.5 (18.6)	53.2 (6.3)	44.0 (7.7)	40.3 (7.5)	15.8 (6.1)	197 (49)	1.14 (0.60)
	GRMD	1.02 (0.24)	3.72 (1.04)	0.37 (0.09)	1.35 (0.27)	2.70 (0.70)	44.1 (19.2)	43.4 (20.7)	45.7 (9.9)	34.7 (10.7)	11.2 (13.1)	209 (56)	2.61 (0.73)
	p	p=0.0010	p=0.0054	p<0.0001	p=0.0001	NS	p=0.0017	NS	NS	NS	NS	NS	p=0.0031
3	Healthy	1.83 (0.23)	4.76 (0.54)	0.76 (0.07)	1.94 (0.15)	2.42 (0.14)	93.4 (15.6)	43.5 (2.8)	42.5 (10.4)	44.6 (11.1)	12.9 (1.9)	222 (25)	1.27 (0.51)
	GRMD	1.2 (0.53)	3.33 (1.43)	0.46 (0.14)	1.28 (0.37)	2.50 (0.61)	38.9 (18)	32.4 (6.7)	52.4 (10.3)	32.2 (8.0)	15.4 (6.9)	195 (67)	3.09 (0.68)
	p	p=0.0145	NS	p=0.0003	p=0.0041	NS	p<0.0001	p<0.0001	NS	p=0.0147	NS	NS	p=0.0002
3.5	Healthy	1.96 (0.20)	4.48 (0.43)	0.86 (0.06)	1.95 (0.09)	2.30 (0.20)	99.2 (18.8)	43.2 (5.9)	45.6 (3.7)	44.2 (3.8)	10.3 (1.9)	250 (38)	0.84 (0.19)
	GRMD	0.99 (0.40)	2.50 (0.97)	0.42 (0.13)	1.07 (0.28)	2.27 (0.52)	25.7 (10.1)	26.1 (3.4)	47.3 (12.2)	32.8 (8.2)	19.9 (11.6)	183 (65)	3.22 (0.94)
	p	p<0.0001	p=0.0002	p<0.0001	p<0.0001	NS	p<0.0001	p<0.0001	NS	p=0.0059	NS	p=0.0340	p<0.0001
4	Healthy	2.06 (0.27)	4.63 (0.77)	0.90 (0.08)	1.93 (0.17)	2.31 (0.12)	96.4 (14.2)	36.7 (5.7)	39.0 (2.9)	49.4 (3.3)	11.6 (1.9)	256 (18)	0.55 (0.20)
	GRMD	1.13 (0.37)	2.73 (1.00)	0.51 (0.07)	1.22 (0.19)	2.21 (0.56)	26.9 (11.8)	23.2 (7.3)	44.7 (10.7)	37.5 (15.3)	17.8 (10.1)	211 (43)	3.17 (0.67)
	p	p<0.0001	p=0.0026	p<0.0001	p<0.0001	NS	p<0.0001	p<0.0001	NS	NS	NS	p=0.0299	p<0.0001
4.5	Healthy	2.13 (0.20)	4.39 (0.32)	0.97 (0.08)	2.01 (0.10)	2.19 (0.11)	97.3 (17.4)	42.3 (7.2)	40.6 (6.6)	50.0 (6.7)	9.4 (4.2)	257 (17)	0.83 (0.29)
	GRMD	1.14 (0.51)	2.54 (1.16)	0.50 (0.09)	1.12 (0.20)	2.20 (0.70)	27.6 (18.4)	21.7 (8.4)	47.7 (9.2)	28.2 (7.8)	24.1 (9.7)	184 (16)	3.73 (0.68)
	p	p<0.0001	p=0.0003	p<0.0001	p<0.0001	NS	p<0.0001	p<0.0001	NS	p=0.0002	p=0.0237	p=0.0031	p<0.0001
5	Healthy	2.27 (0.23)	4.33 (0.37)	1.02 (0.12)	1.94 (0.17)	2.23 (0.03)	105.6 (22.4)	36.7 (4.5)	41.1 (1.4)	47.9 (3.9)	11.0 (3.4)	266 (57)	0.83 (0.12)
	GRMD	1.06 (0.51)	2.26 (1.09)	0.51 (0.15)	1.10 (0.28)	1.97 (0.60)	25.9 (20.7)	21.1 (9.2)	44.1 (9.3)	32.8 (11.2)	23.1 (11.8)	184 (46)	3.54 (1.07)
	p	p=0.0008	p=0.0034	p<0.0001	p<0.0001	NS	p<0.0001	p=0.0005	NS	p=0.0241	NS	p=0.0146	p=0.0003
5.5	Healthy	2.23 (0.09)	4.18 (0.32)	1.05 (0.04)	1.96 (0.13)	2.13 (0.09)	96.6 (16.4)	40.1 (7.0)	40.4 (3.3)	48.5 (3.2)	11.1 (3.2)	280 (49)	0.77 (0.19)
	GRMD	1.01 (0.39)	2.12 (0.82)	0.49 (0.11)	1.03 (0.18)	2.02 (0.56)	28.1 (18.7)	25.7 (8.6)	39.6 (14.9)	41.8 (16.8)	18.6 (5.0)	214 (49)	3.33 (0.68)
	p	p<0.0001	p<0.0001	p<0.0001	p<0.0001	NS	p<0.0001	p=0.0012	NS	NS	p=0.0074	p=0.0280	p<0.0001
6	Healthy	2.38 (0.25)	4.36 (0.32)	1.12 (0.09)	2.05 (0.10)	2.12 (0.15)	90.8 (14.4)	49.4 (5.3)	36.2 (6.2)	54.9 (2.8)	8.9 (4.8)	278 (33)	0.75 (0.34)
	GRMD	1.10 (0.36)	2.25 (0.78)	0.51 (0.08)	1.04 (0.16)	2.14 (0.51)	29.1 (17.3)	24.6 (7.2)	41.8 (9.3)	38.2 (8.9)	19.8 (7.1)	224 (29)	3.26 (0.43)
	p	p<0.0001	p=0.0001	p<0.0001	p<0.0001	NS	p<0.0001	p=0.0039	NS	p=0.0021	p=0.0119	p=0.0101	p<0.0001
6.5	Healthy	2.49 (0.20)	4.41 (0.41)	1.14 (0.07)	2.03 (0.12)	2.18 (0.09)	108.3 (9.8)	40.4 (6.1)	42.9 (3.7)	46.7 (3.6)	10.4 (4.0)	260 (29)	0.63 (0.10)
	GRMD	1.09 (0.40)	2.18 (0.79)	0.54 (0.17)	1.08 (0.28)	2.02(0.44)	26.7 (14.8)	23.8 (6.5)	38.6 (13.0)	40.1 (14.2)	21.2 (7.4)	227 (36)	3.30 (0.43)
	p	p<0.0001	p<0.0001	p<0.0001	p<0.0001	NS	p<0.0001	p<0.0001	NS	NS	p=0.0073	NS	p<0.0001
7	Healthy	2.69 (0.17)	4.72 (0.46)	1.20 (0.09)	2.10 (0.18)	2.25 (0.08)	109.4 (11.1)	46.7 (4.1)	45.8 (6.3)	45.6 (4.5)	8.6 (2.4)	273 (27)	0.72 (0.10)
	GRMD	1.05 (0.32)	2.05 (0.57)	0.53 (0.13)	1.04 (0.18)	1.97 (0.49)	22.9 (12.0)	21.0 (6.8)	36.7 (11.7)	37.7 (9.8)	25.5 (7.1)	222 (36)	3.62 (0.51)
	p	p<0.0001	p<0.0001	p<0.0001	p<0.0001	NS	p<0.0001	p=0.0003	NS	NS	p=0.0011	p=0.0345	p<0.0001
7.5	Healthy	2.64 (0.29)	4.56 (0.54)	1.20 (0.07)	2.07 (0.14)	2.19 (0.15)	100.8 (19.2)	46.8 (5.8)	42.6 (10.2)	47.8 (9.0)	9.7 (3.4)	271 (46)	1.14 (0.53)
	GRMD	0.87 (0.34)	1.68 (0.51)	0.50 (0.18)	0.95 (0.27)	1.75 (0.10)	19.9 (12.4)	21.4 (7.3)	36.3 (19.7)	42.6 (15.1)	21.1 (7.5)	220 (46)	3.68 (0.56)
	p	p<0.0001	p<0.0001	p<0.0001	p<0.0001	p<0.0001	p<0.0001	p=0.0007	NS	NS	p=0.0049	NS	p<0.0001
8	Healthy	2.73 (0.22)	4.68 (0.35)	1.26 (0.04)	2.15 (0.07)	2.17 (0.11)	108.2 (16.8)	49.3 (6.8)	43.5 (8.8)	45.6 (9.2)	10.9 (2.9)	278 (55)	1.11 (0.40)
	GRMD	0.81 (0.31)	1.55 (0.51)	0.46 (0.15)	0.88 (0.23)	1.73 (0.21)	19.8 (14.8)	22.2 (8.2)	44.8 (16.2)	32.6 (11.7)	22.7 (8.1)	222 (37)	3.88 (0.49)
	p	p<0.0001	p<0.0001	p<0.0001	p<0.0001	p=0.0006	p<0.0001	p=0.0008	NS	NS	p=0.0056	p=0.0427	p<0.0001
8.5	Healthy	2.64 (0.33)	4.45 (0.48)	1.21 (0.11)	2.03 (0.16)	2.19 (0.16)	104.2 (20.3)	47.9 (7.6)	40.6 (6.5)	47.1 (6.5)	12.3 (3.1)	290 (48)	1.04 (0.23)
	GRMD	0.92 (0.34)	1.75 (0.53)	0.50 (0.19)	0.94 (0.27)	1.86 (0.40)	19.5 (11.2)	19.9 (7.2)	36.3 (15.6)	38.8 (11.2)	24.9 (8.9)	231 (28)	3.58 (0.64)
	p	p<0.0001	p<0.0001	p<0.0001	p<0.0001	NS	p<0.0001	p=0.0003	NS	NS	p=0.0071	p=0.0198	p<0.0001
9	Healthy	2.61 (0.18)	4.39 (0.26)	1.18 (0.03)	1.99 (0.06)	2.21 (0.14)	111.4 (16.3)	41.7 (2.6)	47.8 (4.8)	43.3 (3.9)	8.9 (3.5)	269 (44)	0.80 (0.24)
	GRMD	0.88 (0.46)	1.66 (0.75)	0.49 (0.22)	0.92 (0.31)	1.77 (0.43)	20.8 (16.9)	20.4 (9.3)	44.7 (13.5)	29.4 (6.6)	23.8 (9.1)	225 (31)	3.7 (0.93)
	p	p<0.0001	p<0.0001	p<0.0001	p<0.0001	p=0.0337	p<0.0001	p=0.0002	NS	p=0.0009	p=0.0033	NS	p<0.0001
Age effect	Healthy	p<0.0001 (↗)	p=0.0024 (↘)	p<0.0001 (↗)	NS	p<0.0001 (↘)	NS	NS	NS	NS	p=0.0089 (↘)	p=0.0145 (↗)	p=0.0068 (↘)
	GRMD	NS	p=0.0067 (↘)	NS	NS	p=0.0350 (↘)	NS	p=0.0034 (↘)	NS	NS	NS	NS	NS
	Group effect	p<0.0001	p=0.0003	p=0.0002	p=0.0002	NS	p<0.0001	p=0.0006	NS	p=0.0427	p=0.0162	p=0.0090	p<0.0001
Correlation with motor score		-0.4353	-0.648		-0.6834	-0.5082	-0.564	-0.5368	-0.3492		0.4454		0.6248
	p	p=0.0002	p<0.0001	NS	p<0.0001	p<0.0001	p<0.0001	p<0.0001	p=0.0030	NS	p=0.0001	NS	p<0.0001
